# MPLA case: I didn't realize those were the expectations!

**DOI:** 10.1002/acm2.14089

**Published:** 2023-07-06

**Authors:** Samantha J. Simiele, Serdar Charyyev, Liyong Lin, Leonard Kim, Dongxu Wang, Mary P. Gronberg, MPLA Cases Subcommittee

**Affiliations:** ^1^ Department of Radiation Physics The University of Texas MD Anderson Cancer Center Houston Texas USA; ^2^ Department of Radiation Oncology – Radiation Physics Stanford University Stanford California USA; ^3^ Department of Radiation Oncology Emory University School of Medicine Atlanta Georgia; ^4^ Department of Radiation Oncology MD Anderson Cancer Center at Cooper and Cooper Medical School of Rowan University Camden New Jersey USA; ^5^ Department of Medical Physics Memorial Sloan Kettering Cancer Center New York New York USA; ^6^ Department of Radiation Physics The University of Texas MD Anderson Cancer Center and UT Health Houston Graduate School of Biomedical Sciences Houston Texas USA

**Keywords:** adaptability, case study, communication, conflict management, mentorship, MPLA

## Abstract

This work of fiction is part of a case study series developed by the Medical Physics Leadership Academy (MPLA). It is intended to facilitate the discussion of how students and advisors can better communicate expectations and navigate difficult conversations. In this case, a fourth‐year Ph.D. student Emma learns that her advisor Dr. So is leaving the institution and has not arranged to bring any students with him. As Emma and Dr. So meet to discuss Emma's next steps, the conversation reveals misunderstandings and miscommunications of expectations, including a specific publication requirement for graduation from Dr. So. Having just learned of Dr. So's publication requirement, Emma realizes that graduating before the lab shuts down is not feasible. The intended use of this case, through group discussion or self‐study, is to encourage readers to discuss the situation at hand and inspire professionalism and leadership thinking. This case study falls under the scope of and is supported by the MPLA, a committee in the American Association of Physicists in Medicine (AAPM).

## 1 INTRODUCTION AND NARRATIVE

*This work of fiction is part of a case study series developed by the Medical Physics Leadership Academy (MPLA). The intended use of this case, through group discussion or self‐study, is to encourage readers to discuss the situation at hand and inspire professionalism and leadership thinking*.

*While inspired by real‐life events, the characters, locations, and institutions in this case study have been modified. Name, gender, and other identities (if perceived) in this case are only representative. The facilitators of the case are welcome to change any identities for their educational purposes*.

*This case study falls under the scope of and is supported by the MPLA, a committee in the American Association of Physicists in Medicine (AAPM)*.

Emma is a fourth‐year graduate student in Dr. So's research lab. Her project was, according to Dr. So, one of the most challenging he had supervised to date. The bench techniques required to complete the experiments were difficult to learn and even harder to master. But having recently published her first manuscript and having passed her candidacy exam, Emma was proud of what she'd accomplished so far.

Emma's most recent series of experiments had left her exhausted. She had spent several weeks working around the clock collecting and analyzing data, and she had arrived at a difficult conclusion: the results did not support her and Dr. So's hypothesis. Either a mistake had been made in the experiments, and they would have to be repeated, or the project was taking a turn, and another direction needed to be investigated.

Emma headed home for the evening with her thoughts on her findings. She wished she could talk with her previous lab mates, all of whom had recently graduated. She could use their advice on how to ask Dr. So about next steps.

At home, Emma settled into her couch with a cup of Chai tea and opened her email. She usually ended her day responding to any emails she was unable to answer while performing experiments. She was surprised to see a new email from Dr. So. She had not heard from him in over a month due to his busy travel schedule. Emma opened the email, and her heart raced.

TO: Emma Daniels

FROM: Andrew So

SUBJECT: Need to meet

The lab is closing in 6 months. Please schedule a meeting with me. Need to discuss your plans.

Emma reread the email several times and was overcome with emotion. She had so many questions. Why is the lab closing? What "plans" are Dr. So referencing? I'm finishing my degree! I've been here for three and a half years; I need to finish. I can't start over. I won't start over. Maybe he'll allow me to graduate. Maybe that's what he wants to discuss. Could I graduate in 6 months at the end of my fourth year?

Emma decided she would respond to Dr. So's email and ask for a meeting on Friday, 2 days from now. The buffer would provide her with time to strategize and develop talking points. Although, in this situation, Emma wasn't sure what talking points she should be developing. Dr. So had always been brief in his emails, but this time his brevity left Emma wondering if she should be developing a plan to finish her degree in 6 months or an argument for… something else?

Dr. So responded, letting Emma know he could meet with her at 2:00 PM this Friday.

The next 2 days were challenging for Emma. She was anxious and found it difficult to focus on work. She spent Thursday outlining her plan to finish her degree in 6 months. She realized how challenging that would be. She was familiar with long hours and poor work‐life balance but was not sure how much longer she could operate with so little sleep.

**LEARNING OBJECTIVES (LO)†**

1. Recognize the value of transparency and communication skills to convey clear expectations with your boss/employees.
2. Discuss strategies for conflict management.
3. Describe the role of adaptability in unexpected situations.
4. Appraise the value of emotional self‐awareness and emotional self‐control in navigating difficult conversations.
5. Develop organizational awareness and examine contractual, formal or informal professional relationships in the setting of a graduate program and in a general workplace.
6. Identify aspects of responsible behavior for supervising/mentoring others.

Emma reviewed her notes one last time and then walked up the stairs to the faculty offices on the second floor. She approached Dr. So's office door, took a deep breath to help calm her nerves, and knocked. She was met with an indifferent voice asking her to come in. Emma opened the door to find Dr. So at his desk, typing rapidly. He motioned for her to take a seat. Emma sat down and waited silently for several minutes until he finished typing. He then turned to her and asked: “Well, you received my email. What questions do you have?”

For a moment, Emma was speechless, and then she managed to say, “Your email indicated the lab was closing. Could you share more information about the situation?”

Dr. So sighed and said, “I'm moving to a new institution in six months, and I have not made arrangements to bring graduate students with me.”

This meant for Emma to graduate she would need to complete the work for her degree and meet the graduation expectations in a very limited time. She felt a sense of hope and told Dr. So, “I have met the publication requirements of both the graduate school and the department by having published a first‐authored manuscript. I think I can finish my degree before you leave.”

Dr. So shook his head and said, “But you haven't met the lab requirement of three first‐authored publications. What is the status of your dissertation work? Will you be able to submit two first‐authored publications and have your dissertation written and defended within six months?”

Emma felt caught short. Dr. So's publication expectation had never been communicated to her. Emma mustered her courage and decided to advocate for herself. “The graduate school requirement is one first‐authored paper. I wasn't aware of a three‐paper requirement. Considering I wasn't aware of this and that my project requires advanced bench techniques, more so than most projects, can we compromise on two first‐authored publications?”

Dr. So frowned and stated, “I will not lower my standards for any student.”

Silence followed as Emma considered how to proceed. “Dr. So, when I joined the department, I was informed about the publication requirements of the graduate school and the department. This is the first time I'm hearing that you require three publications. How is it possible that I was unaware of this expectation after working for you for more than three years?”

Dr. So seemed displeased. “How would I know? All my other students had at least three first‐author papers before graduation, which you would have easily known if you knew their work. I know you spent time with them and talked to them before they graduated. It seems to me this was certainly an oversight on your part.”

Taken aback, Emma continued, “What about the mentor/mentee agreement we signed when I started in the lab? That included your expectations, and there's nothing about this in there.”

Dr. So shook his head and explained, “The mentoring agreement is a graduate school formality. I had nothing to do with what's in that form.”

Emma responded with a determined tone: “Okay, well, I'll just get it done. I'll figure it out.”

Dr. So's response did not convey as much faith as Emma had, “Even working around the clock, that will be very difficult. I don't think you will be able to produce two quality manuscripts and finish your dissertation in six months. Do you have a backup plan?”

Emma's frustration was starting to show. She asked, “If I can't graduate, what options are available to me?”

Calmly, Dr. So replied, “I think it would help me to understand your long‐term career goals.”

Emma shared, “I am keeping my options open. A Ph.D. would allow me to pursue a variety of career paths.”

Dr. So seemed displeased again. “It's disappointing to me that almost four years into your degree, you still don't know what you want to do after graduation. All of my students have pursued a career in industry.”

Emma explained, “I do have an interest in industry. However, I think patient care and a clinical role could also suit me, and I want to keep that option open.”

Dr. So was finished with the conversation. “Emma, I think we're done here. It sounds like you need to figure out your life. How about you come back to me when you know what you want. I need an answer by the end of next week so I can help guide you during our remaining time together.”

Emma felt defeated. She thanked Dr. So and left his office.

## 2 DISCUSSION

This case is in the scenario of *Problem Diagnosis* according to the Case Study Handbook by Ellet.^1^

The following are suggested discussion questions in relation to the Learning Objectives (LO):

1. What contributing factors led to Emma being unaware of Dr. So's publication requirement? How could this misunderstanding have been avoided? (LO1, LO5)
2. Should students and their advisor(s) sign mentorship agreements? If agreements are used, what information should be included? (LO1, LO5)
3. How did Emma prepare for her meeting with Dr. So? What other strategies could she use to prepare for this meeting? (LO1, LO4)
4. Do you agree with how Emma handled herself in her discussion with Dr. So? Was it appropriate for Emma to try to compromise with Dr. So on a publication requirement of two papers? (LO2, LO4)
5. Do you agree with how Dr. So handled himself in his discussion with Emma? Is it fair for him to be frustrated with his student? (LO4, LO6)
6. If you were in Emma's shoes, what would you do next? (LO3, LO5)

Sample answers to the suggested discussion questions are available in the Supplemental Materials, or by writing to the MPLA Cases Subcommittee: (https://www.aapm.org/org/structure/default.asp?committee_code=MPLACA)

## 3 CONCLUSION

Case study participants should leave the discussion with an appreciation for the Learning Objectives chosen for this case study session and consider the real‐life applications in their own workplace and educational setting.

## AUTHOR CONTRIBUTIONS

Samantha J. Simiele, Serdar Charyyev, and Mary P. Gronberg drafted the initial case text. Liyong Lin, Leonard Kim, and Dongxu Wang provided critical revisions to the case text. All authors contributed to the learning objectives and approved the final version.

## CONFLICTS OF INTEREST STATEMENT

The authors declare no conflicts of interest.

## Supporting information

Supporting Information

## Footnotes

† The underscored terms are key words from MPLA Curriculum. Additional material and resources on those topics can be found on the MPLA Curriculum at https://w3.aapm.org/leadership/curriculum.php. We encourage readers to review this material as part of the exercise.
