# Supplementary material for: MPLA case: I didn't realize those were the expectations!
Source: J Appl Clin Med Phys. 2023 Jul 6;24(8):e14089. doi: 10.1002/acm2.14089 (PMC10402668; doi:10.1002/acm2.14089)
Supplement: Supplementary file 1 — Supporting Information [file ACM2-24-e14089-s001.docx]

**Supplemental Materials**

*Sample answers to the suggested discussion questions:*

1. The omittance of the publication requirement in the mentorship agreement as well as Emma and Dr. So’s infrequent communication led to Emma’s oblivion of Dr. So’s publication requirement. To avoid this misunderstanding, Emma should have clarified with Dr. So what his expectations for her are, including graduation requirements. This discussion should have happened before signing the mentorship agreement.

Furthermore, consistent communication with Dr. So could have prevented potential misunderstandings. Creating a timeline of her experiments and expected degree completion and reviewing it with Dr. So regularly would have revealed this misunderstanding. It can be difficult for new graduate students to navigate or even recognize the importance of these discussions at such an early stage in their careers. Dr. So. should have included his expectations in the mentorship agreement template provided by the graduate school or been prepared to separately discuss his expectations. Additionally, many advisors have busy travel schedules, but being available for regular meetings with graduate students is a typical requirement of an advisor, and Dr. So’s absence impacted regular communication.

Another aspect that can be reflected upon by both Emma and Dr. So is organizational awareness, especially regarding informal relationships, unspoken rules, or hidden cultural expectations in a workplace. From the case narrative, Dr. So thinks Emma should at least have known about and was expected to match the publication record of previous graduates, yet Emma was not aware or did not make that connection. Misunderstandings and miscommunications due to unspoken rules or hidden culture are rather typical situations in any social environment. The established and senior members should help novices of the group to comprehend such nuances, while a new member may need to be extra sensitive in assessing them. A Podcast link^3^ is provided as a reference to this topic.

Another related topic should also be discussed, that is the relationship between a graduate student, his/her funding or non-funding mentor(s), as well as the graduate program itself. Even if Dr. So’s expectation were clear to Emma, she might not be able to graduate within 6 months from the time point of this event anyway, before her PI’s departure from this institution. What is Emma’s formal status in this graduate program after Dr. So leaves? Will Dr. So still have any say regarding her graduation? What will be the graduate school’s responsibility to a student when the PI departs? Each academic institution or graduate program may have different settings, but the general organizational awareness for any contractual and binding relationship between a graduate school, a faculty, and a student can assist in resolving the difficult situation faced by Emma.

1. Mentorship agreements can be beneficial to both advisors and students, as they can help align expectations and prevent miscommunication. Mentorship agreements should include both the advisor’s expectation of his/her students and what the student can reasonably expect from his/her advisor. There are many mentorship examples and templates available on the web. Mentorship agreements can include topics such as research expectations, communication standards, meeting frequency, conference attendance, publication requirements, and participation in lab events.
2. It is particularly challenging to prepare for a meeting when you are not aware of the meeting’s intent. In this scenario, Emma was not sure what Dr. So meant by “Need to discuss your plans”. She assumed that Dr. So wanted to discuss how she was going to graduate in six months and prepared accordingly. It would have been beneficial for her to brainstorm other things that Dr. So could want to discuss and prepare for those scenarios. Additionally, she could have given some thought to what she would do if Dr. So does not agree with her plan. Dr. So could have assisted Emma in preparing for her meeting by providing greater transparency and information in his original email.
3. Overall, Emma handled herself well during her meeting with Dr. So. She remained calm and collected throughout the conversation. In this situation, it was reasonable for Emma to try to negotiate with Dr. So since his mentorship and direction had been scarce. However, before negotiating with him, she could have tried to work together with him to further understand her options for moving forward.
4. Dr. So was rather disrespectful during this conversation. He should have acknowledged how important and difficult this decision is for Emma and provided his full attention as soon as she arrived at his office. He should also have been emotionally self-aware that he was displeased with Emma’s lack of understanding of his graduation expectation, but he needed to accept some responsibility for their miscommunication. Moreover, he needed to recognize that each student is unique, and Emma’s goals may be different from those of his previous students.
5. If I were in Emma’s shoes, I would look to see if there are resources at the graduate school or institution for navigating difficult situations, such as an Ombudsman Office. It would be beneficial to receive advice from professionals who are trained in conflict remediation. I would also seek advice from mentors, such as the members of my Ph.D. advisory committee. I would also clarify with the graduate school or the institution regarding the exact relationship and duties a graduate student has with his/her PI vs the graduate program.
